# A seven-year study on the effect of the pre-erythrocytic malaria vaccine candidate RTS,S/AS01 _E_ on blood stage immunity in young Kenyan children

**DOI:** 10.12688/wellcomeopenres.15002.1

**Published:** 2019-03-05

**Authors:** Francis M. Ndungu, Jedida Mwacharo, Juliana Wambua, Patricia Njuguna, Kevin Marsh, Chris Drakeley, Philip Bejon

**Affiliations:** 1 Department of Biosciences, KEMRI/Wellcome Trust Research Programme, Kilifi, 80108, Kenya; 2 Infection & Immunity Department, London School of Hygiene & Tropical Medicine, London, WC1E 7HT, UK

**Keywords:** Plasmodium falciparum, malaria, RTS, S/AS01 E, Vaccines, immunity, pre-erythrocytic, blood stages

## Abstract

**Background**: RTS,S/AS01 _E_, the most advanced malaria vaccine confers partial immunity. The vaccine-induced pre-erythrocytic immunity reduces exposure to blood-stage parasites, delaying acquisition of antibodies to blood-stage antigens.  However, the duration of this effect is unknown.

**Methods:** We measured, by enzyme-linked immunosorbent assay, IgG-antibodies to 4 *Plasmodium falciparum *blood-stage antigens (AMA1, MSP1 _42_, EBA175, and MSP3) on 314 children randomized to receive RTS,S/AS01 _E_ or Rabies vaccine at 5 – 17 months of age in a phase 2b trial in Kenya, and thereafter participated in a 7-year study of the duration of vaccine immunity.

**Results**: Antibody levels to MSP1 _42_, AMA1 and EBA175 were slightly lower among the RTS,S/AS01 _E_ recipients, relative to the Rabies-control vaccinees, during the first 48 months of surveillance. Irrespective of vaccine arm, antibody levels to merozoite antigens were positively associated with the risk for malaria. However, this was only apparent at high levels for EBA175 and AMA1 and was not evident after adjusting for heterogeneity in malaria-exposure. Among children with asymptomatic parasitaemia, antibody levels were associated with reduced clinical malaria.

**Conclusions**: The reduction in levels of antibodies to blood-stage antigens induced by vaccination with RTS,S/AS01 _E_ can last for several years. In absence of asymptomatic infection, anti-merozoite antibody levels were unreliable correlates of clinical immunity.

## Introduction

Despite the recent gains in malaria control, the disease remains a major public health risk, with 216 million cases and 445,000 deaths associated with malaria in 2016 ^1^. Progress in malaria control has stalled and may have reversed in some areas ^2^.

RTS,S/AS01 _E_ is the most advanced candidate malaria vaccine and is based on the circumsporozoite protein (CSP) that targets the pre- erythrocytic cycle of *Plasmodium falciparum* in humans. Vaccination with RTS,S/AS01 _E_ has been partially efficacious against malaria in phases II and III trials in Africa ^3, 4^. RTS,S/AS01 _E_ induces pre-erythrocytic immunity. In contrast, naturally acquired immunity to malaria is largely dependent on antibodies to blood-stage parasites including the merozoite stage. Although there are no unambiguous correlates of natural immunity ^5^, antibodies to merozoite antigens have been associated with protection through multiple mechanisms including the inhibition of erythrocyte invasion and replication ^6^, complement-dependent mechanisms ^7^, and enhancement of uptake and clearance by circulating phagocytes ^8, 9^. Antibodies to antigens expressed on the surface of infected red blood cells (iRBCs) have also been associated with immunity, which could inhibit or reverse sequestration of iRBCs, inhibit formation of rosettes, and promote opsonization of iRBCs for uptake by phagocytes ^10– 12^.

Antibodies to malaria parasites are acquired as a result of exposure. As such, interventions like insecticide impregnated bed nets and RTS,S/AS01 _E_ -vaccination that reduce exposure to blood-stage antigen will affect the rate at which antibodies to merozoite and other blood-stage antigens are acquired. Previously, we and others demonstrated that RTS,S/AS01 _E_ and RTS,S/AS02 vaccinations reduced blood stage antibody levels, likely as a result of reducing the exposure to blood stage parasites due to induction of partial pre-erythrocytic immunity ^13, 14^. However, the duration of this effect remains unknown. It is important to determine the duration of this effect as RTS,S/AS01 _E_ vaccination could delay the development of naturally acquired immunity, increasing the possibility of continued susceptibility in older children after the waning of the vaccine induced immunity ^15^.

In this study, we aimed to determine the durability of the previously reported reduction in antibody levels to merozoite antigens in children receiving RTS,S/AS01 _E_ vaccination, relative to Rabies control vaccines ^13^. We analysed plasma samples collected from children during a seven-year extended follow up of a phase IIb randomized, controlled trial of RTS,S/AS01 _E_ among young children in Kilifi, Kenya, examining antibodies to 4 different merozoite antigens by enzyme-linked immunosorbent assay (ELISA). We then analysed the effect of RTS,S/AS01 _E_ vaccination on the acquisition of these antibodies and tested for potential correlations between antibody levels and protection from clinical malaria episodes.

## Methods

### Study design

447 healthy Kenyan children aged 5 – 17 months were randomized in a 1:1 ratio to receive 3 doses at monthly intervals of either RTS,S/AS01 _E_ or Rabies vaccine in a phase 2b trial, to evaluate the efficacy and safety of RTS,S/AS01 _E_ against clinical malaria episodes by *P. falciparum* infection. Details have been published elsewhere ^3^.

### Monitoring for episodes of clinical malaria

The primary end point was a clinical episode of malaria, defined as an axillary temperature of >37.5°C, with a *P. falciparum* parasite density of 2500 parasites/microlitre of blood. Active surveillance was implemented with weekly home visits, where children were screened for fevers associated with *P. falciparum* parasites, both during the trial and the extended follow up period. A parallel passive surveillance was implemented by field workers residing in the study villages and health care staff in local health facilities.

Asymptomatic infections were detected by both microscopy and blood-smears during the cross-sectional data and sample collecton surveys described below.

### Blood samples

Vaccines doses were given at month 1, 2 and 3. Blood samples were initially taken (1) before vaccination (in March 2007), (2) 1 month after dose 3, (3) in March 2008 (i.e., mean, 8 months; range, 4–10 months after dose 3), and (4) 12 months after dose 3. Subsequently, the study was extended to test the duration of vaccine induced immunity, and further blood samples were collected in March (5) 2009, (6) 2010, (7) 2011, (8) 2012, (9) 2013, and (10) 2014. Separated plasma was aliquoted and stored at 80°C until assayed.

Previously, we reported anti-merozoite antibody responses for the samples collected from four time points during the first 14 months of follow up ^13^. In the current study, we extend the analysis to samples collected during the extended study, including 10 timepoints taken over 7 years; i.e. pre-vaccination (i.e. month 0), then at 4, 6.5, 8, 14, 24, 36, 48, 60, 72, and 84 months.

### ELISA

Samples were tested by ELISA for the presence of human IgG against the following *P. falciparum* antigens as described elsewhere ^16^: MSP1 _42_, 3D7 sequence expressed in *Escherichia coli* ^17^; MSP3, FVO sequence, expressed in *E. coli* ^18^; the receptor-binding domain II (PfEBA175RII) of EBA175, 3D7 sequence, expressed in *P. pastoris* ^19^; and AMA1, 3D7 sequence expressed in *E. coli* ^20^. In brief, each antigen was coated onto high absorbance plates (Immulon4 HBX) at a con- centration of 0.5 micrograms/mL and stored at 4°C overnight. The plates were washed 3 times in phosphate-buffered saline (PBS) with 0.05% Tween 20 (PBS-T) and blocked for 3 h with blocking buffer (1% w/v dried skimmed milk powder in PBS-T). After 3 additional washes, 100 microlitre of each plasma sample were added to duplicate wells at a final dilution of 1/1000 in PBS-T. The next day, after 5 washes, 100 microlitre of horse radish peroxidase–conjugated antihuman IgG (DAKO) at a dilution of 1:5000 in blocking buffer was added to each well, and plates were incubated for 3 h. The plates were then developed using H _2_0 _2_ as substrate and OPD (Sigma) as the colorimetric indicator for 20 min in the dark. Plates were read at 492 nm on a Molecular Devices Versa Max ELISA reader. Tests were repeated if duplicate optical density (OD) values for an individual plasma sample varied by more than a factor of 1.5. A pool of serum samples from an area in Africa where malaria is highly endemic was titrated on each plate and acted both as a positive control and provided values for a standard curve for converting optical density (OD) readings into arbitrary units, minimizing inter-plate and inter-day variations. A 3-parameter sigmoid ligand binding model was used to least-squares fit a curve to the values of the hyperendemic serum sample pool, and this was used to calculate sample antibody levelss on each plate.

### Statistical analysis

Antibody scores from ELISAs were expressed relative to the OD readings obtained from the hyperimmune standard, with a score of 1000 scaled to be the maximum reactivity seen at the lowest dilution used in the hyperimmune standard curve, and then log-transformed before analysis. Student’s T-test with comparison of means and non-parametric analyses with comparisons of medians and rank sum tests were used to compare groups. For the prospective association with malaria risk, the antibody levels were split into deciles, and a Poisson regression analysis was conducted with the unit of analysis being the period of time after each antibody level was estimated, hence including up to 10 observations per child, using the clustered sandwich estimate in Stata 15 (StataCorp LLC). We used the exposure index, as previously described ^21^, to estimate exposure to malaria based on geographical location.

## Results

**Figure 1.**
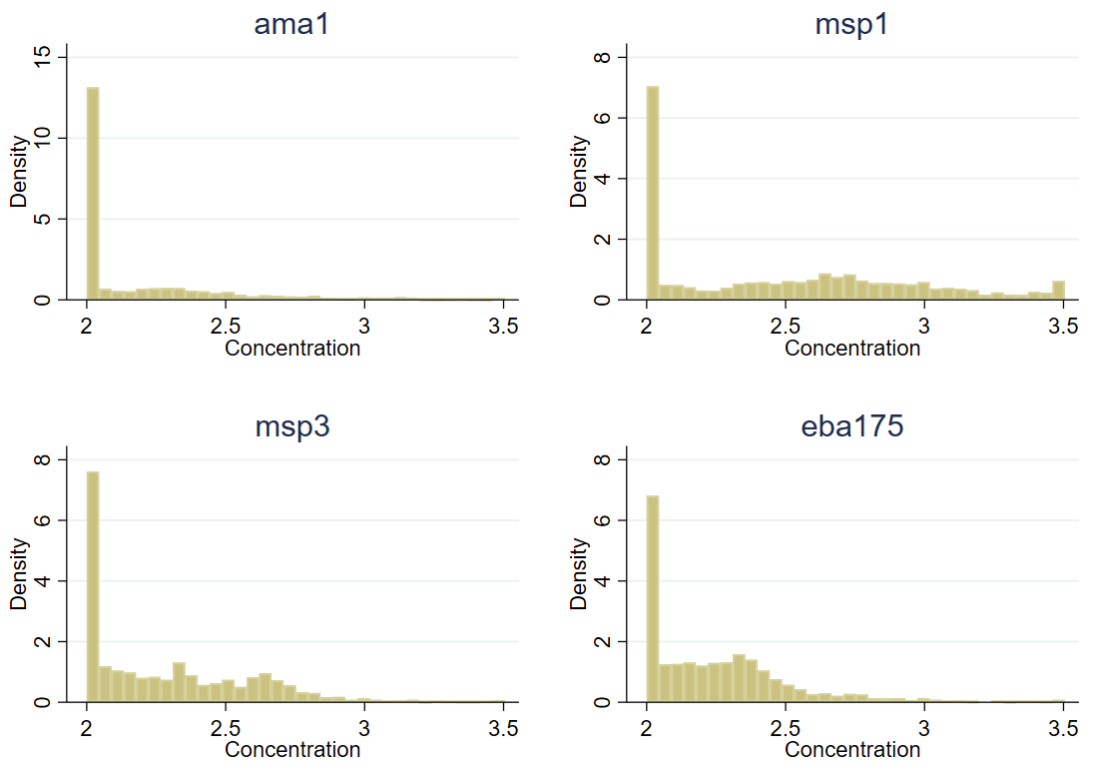
Distribution of anti-merozoite antibody levels. Antibody levels were measured by ELISA.

1735 plasma samples collected from 10 time points: at 0 (i.e. pre-vaccination), 4, 6.5, 8, 14, 24 (March 2009; n = 314), 36 (March 2010; n = 303), 48 (March 2011; n=295), 60 (March 2012; n=276), (72) (March 2013; n = 269), and 84 (March 2014; n=278) months of the third dose of vaccination were tested for antibody levels. The antibody levels varied widely, with the majority of the children being unresponsive (i.e. lower than the lowest value on the straight part of the sigmoid curve based on the dilution of the hyperimmune standard serum), while the rest had values lying within the straight part of the hyperimmune standard curve ( Figure 1).

### Anti-merozoite antigen antibody levels split by RTS,S/AS01 _E_ vaccination

**Figure 2.**
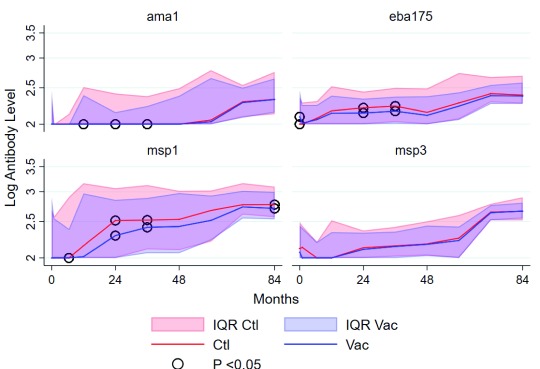
Comparison of the mean levels of anti-merozoite antigen-specific antibodies between RTS,S/AS01 _E_ and Rabies control vaccinees. Antibody levels were determined by ELISA and the values log-transformed to achieve normal distributions. Student’s T-test with comparison of means and non-parametric analyses with comparisons of medians and rank sum tests were used to compare groups. The read blue and red lines indicate the mean levels of RTS,S/AS01 _E_ and Rabies control vaccinations, respectively. The shaded regions indicate the 95% confidence intervals.

Geometric mean antibody levels for all the 4 merozoite antigens increased with age, irrespective of vaccination group, but this was more apparent for 3 of the 4 antigens, and less apparent for AMA1 ( Figure 2). There were indications of seasonal variation during the first year of sampling when 4 samples were collected per child, as previously described ^13^, but it was not possible to assess seasonality once sampling was scaled back to 1 sample per child per year, timed to occur in the dry period just before the main transmission season. Antibody levels for AMA1, EBA175 and MSP1 _42_ diverged after vaccination, with levels being higher among the Rabies control vaccinees than the RTS,S/AS01 _E_ vaccinees at months 12, 24 and 36, 24 and 36, and 6, 12, 24, 36 and 84 of the third dose of vaccination for AMA1, EBA175 and MSP1 _42_, respectively ( Figure 2). Thus, the divergence was temporal for AMA1 and EBA175, as the differences in the median antibody levels reduced with time and were similar by 48 months of the third dose of vaccination. In contrast, anti-MSP1 _42_ antibody levels were still higher among the Rabies-control than the RTS,S/AS01 _E_ vaccinees at 84 months of the third dose (the last time point of sampling) with statistical significance ( Table 1). Similar patterns were seen on non-parametric analyses with medians.

**Table 1.** Comparisons of the geometric mean antibody levels of antibody levels between RTS,S/AS01 _E_ and Rabies control vaccines at different time points.

Antigen	Month	RTS,S/AS01 E	Rabies Control	P value
ama1	0	1.61 (1.56-1.66)	1.66 (1.61-1.71)	0.156
ama1	4	1.56 (1.53-1.59)	1.55 (1.53-1.58)	0.705
ama1	8.5	1.59 (1.56-1.63)	1.55 (1.53-1.58)	0.057
ama1	12	1.67 (1.61-1.73)	1.61 (1.56-1.66)	0.116
ama1	24	1.64 (1.6-1.69)	1.58 (1.55-1.62)	0.031
ama1	36	1.68 (1.62-1.73)	1.62 (1.58-1.67)	0.12
ama1	48	1.7 (1.65-1.76)	1.67 (1.62-1.72)	0.311
ama1	60	1.8 (1.73-1.87)	1.78 (1.71-1.84)	0.628
ama1	72	1.85 (1.8-1.9)	1.83 (1.78-1.88)	0.588
ama1	84	1.92 (1.86-1.98)	1.9 (1.85-1.96)	0.605
eba175	0	1.66 (1.6-1.71)	1.71 (1.66-1.76)	0.114
eba175	4	1.61 (1.58-1.63)	1.61 (1.58-1.63)	0.939
eba175	8.5	1.65 (1.62-1.68)	1.62 (1.6-1.65)	0.208
eba175	12	1.75 (1.69-1.81)	1.69 (1.65-1.73)	0.095
eba175	24	1.74 (1.7-1.79)	1.68 (1.65-1.71)	0.016
eba175	36	1.79 (1.74-1.84)	1.71 (1.68-1.75)	0.008
eba175	48	1.76 (1.7-1.82)	1.68 (1.65-1.72)	0.023
eba175	60	1.88 (1.82-1.95)	1.78 (1.73-1.83)	0.011
eba175	72	1.98 (1.93-2.03)	1.92 (1.88-1.96)	0.068
eba175	84	1.97 (1.92-2.02)	1.94 (1.9-1.98)	0.435
msp1 _42_	0	1.75 (1.67-1.84)	1.81 (1.72-1.9)	0.36
msp1 _42_	4	1.72 (1.65-1.79)	1.7 (1.64-1.76)	0.687
msp1 _42_	8.5	1.81 (1.74-1.88)	1.69 (1.63-1.74)	0.006
msp1 _42_	12	1.99 (1.87-2.11)	1.86 (1.76-1.95)	0.083
msp1 _42_	24	2.08 (1.99-2.16)	1.93 (1.85-2)	0.009
msp1 _42_	36	2.12 (2.04-2.2)	1.99 (1.92-2.07)	0.016
msp1 _42_	48	2.07 (1.99-2.14)	2 (1.93-2.07)	0.186
msp1 _42_	60	2.15 (2.08-2.22)	2.08 (2.01-2.14)	0.116
msp1 _42_	72	2.34 (2.29-2.4)	2.28 (2.23-2.32)	0.069
msp1 _42_	84	2.32 (2.27-2.38)	2.25 (2.2-2.29)	0.019
msp3	0	1.73 (1.67-1.78)	1.7 (1.65-1.75)	0.412
msp3	4	1.71 (1.67-1.75)	1.66 (1.62-1.7)	0.084
msp3	8.5	1.59 (1.56-1.62)	1.58 (1.56-1.6)	0.563
msp3	12	1.7 (1.63-1.76)	1.64 (1.59-1.69)	0.134
msp3	24	1.7 (1.66-1.74)	1.66 (1.63-1.69)	0.191
msp3	36	1.75 (1.7-1.8)	1.7 (1.66-1.74)	0.126
msp3	48	1.75 (1.71-1.8)	1.73 (1.69-1.77)	0.476
msp3	60	1.82 (1.76-1.88)	1.75 (1.7-1.79)	0.062
msp3	72	2.18 (2.13-2.22)	2.16 (2.13-2.19)	0.444
msp3	84	2.2 (2.16-2.24)	2.17 (2.14-2.21)	0.396

### Antibody levels and subsequent risk of clinical malaria

**Figure 3.**
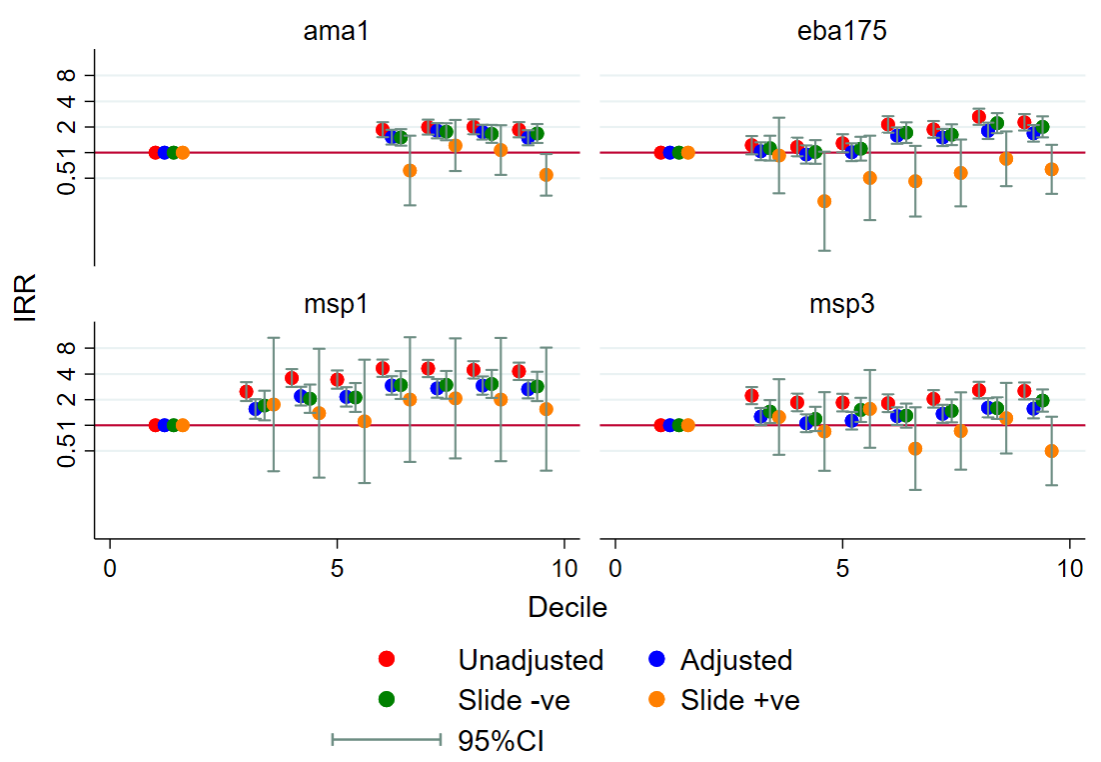
Prospective association of antibodies with immunity to malaria. Antibody levels were split into deciles and tested for association with the numbers of malaria episodes in the ensuing malaria transmission period, but before the next sampling time point. Poisson regression analysis was conducted with the unit of analysis being the period of time after each antibody level was estimated, hence including up to 7 observations per child, using the clustered sandwich estimate. The analysis adjusted for age, exposure index, vaccine arm, and bed net usage. The red and blue dots indicate unadjusted and adjusted analyses. The green and orange dots indicate analysis for parasite negative and positive samples.

Antibody levels were split into deciles, which were then tested for prospective associations with protection from malaria in the transmission period after each, but before, the next sampling time-point. Pre-existing antibody levels for the 4 different merozoite proteins were not associated with clinical immunity ( Figure 3). Instead, the incident rate ratio for clinical malaria increased with rising antibody levels. This relationship was most apparent at higher antibody levels (>5 ^th^ decile) for AMA1 and EBA175 (irrespective of vaccine arm). However, the incident rate ratios for the effect of antibodies on clinical malaria reduced after controlling for heterogeneity in malaria exposure using an exposure index, suggesting that these anti-merozoite antibodies are markers of exposure, rather than immunity.

Furthermore, when all the data were stratified by asymptomatic-parasite positivity at sampling by microscopy, the highest levels for AMA1 and MSP3, and all the of levels for EBA175 above the non-reactive group, were associated with reduced rate ratios for clinical malaria, among the children with asymptomatic parasitaemia at the time of sampling. Associations between higher antibody levels and increased incident rate ratios were maintained among the children without asymptomatic parasitaemia ( Figure 3).

## Discussion

We and others reported previously that RTS,S/AS01 _E_ vaccination resulted in a reduction in antibody levels to blood stage malaria antigens ^13, 14^. However, the duration of this effect is unknown. Here, we investigated the longevity of the reduction of antibody levels to four blood stage antigens after an extended follow up of the vaccines and controls for up to 7 years post-vaccination. We found that immunization with RTS,S/AS01 _E_ and the associated clinical protection resulted in the reduction of antibody response to MSP1 _42_, AMA1 and EBA175 antibody levels but not for MSP3. While the antibody levels for AMA1 and EBA175 among RTS,S/AS01 _E_ vaccinees were below those measured in the Rabies control vaccinees during the first 48 months of monitoring, antibodies to MSP1 _24_ remained lower in the RTS,S/AS01 _E_ vaccinees than in the controls throughout the study period. This latter, persistent difference was statistically significant except at the very last timepoint, when statistical significance was only marginal, considering that there are multiple comparisons by timepoint and by adjuvant (p=0.019).

In this study, antibody levels to four specific merozoite antigens were not associated with clinical protection. Rather anti-merozoite antibody levels were positively associated with the risk of clinical malaria for the group as a whole. The most likely explanation for this is that antibody responses are markers of exposure, and therefore represent ongoing risk of future exposure to malaria, and this interpretation is supported by the fact that the positive association was reduced after controlling for the exposure index. It is possible that higher antibody titers might have been protective (i.e. those above a protective threshold ^22^). In the presence of asymptomatic infection, anti-EBA175 antibodies at all the deciles were higher than the lowest (i.e. non-reactive) decile, and some of the higher deciles for AMA1 and MSP3 antibodies were associated with protection from clinical malaria (irrespective of the vaccine arm). This finding is consistent with several previous studies where analyses of single antigen-specific antibody responses within whole populations demonstrated no protective effect of antimalarial antibodies, but the same antibodies were associated with clinical immunity when parasite-positive individuals were analysed separately ^22, 23^. Our analysis involves only four antigens and there is evidence that the breadth of antibody positivity is also important for protection ^24, 25^.

An RTS,S/AS01 _E_ induced reduction in blood stage immunity will have implications for the outcomes of vaccination if the vaccine is deployed for routine use among African children. If vaccination resulted in delayed development of natural immunity, then some of the gains of the vaccination may be offset by delayed susceptibility as the vaccine induced immunity wears off. Studies done to date on Phase II trials have suggested this possibility with a three-dose vaccine regimen ^15^, although the effect may be countered by a fourth dose ^26^. We show here that antibodies to blood stage immunity are reduced after vaccination with RTS,S/AS01 _E_, which is consistent with induction of pre-erythrocytic immunity leading to a reduced incidence of blood-stage parasitaemia. However, antibodies induced by natural exposure to the four blood stage antigens tested were not consistently associated with immunity to malaria and there is no widely accepted or consistent immunological marker for immunity to malaria. It will be important to combine RTS,S/AS01 _E_ with other malaria control measures like insecticide treated nets for protecting individuals from malaria, and further clinical evaluations of the four-dose vaccine regimen should include long-term follow up in the implementation trials.

## Data availability

### Underlying data

Havard Dataverse: Replication Data for: Effect of rtss vaccination on blood stage immunity data, https://doi.org/10.7910/DVN/KH9ESP ^27^.

Data are available under the terms of the Creative Commons Attribution 4.0 International license (CC-BY 4.0).

## Ethical considerations

The study protocol and its subsequent amendments received ethical and scientific approval from the Kenyan Medical Research Institute National Ethics Committee. The study was overseen by an independent data-monitoring committee and local safety monitors and was conducted in accordance with the Helsinki Declaration of 1964 (revised 1996) and Good Clinical Practice guidelines. Written informed consent in the local languages (Swahili or Giriama) was required from parents/guardians for participation.

## Author information

FMN and PB conceptualized the study, supervised and managed the collection of immunology data, analysed and interpreted the data, and wrote the paper. JM performed the antibody measurements, JW conducted and supervised surveillance for malaria, and sample collection, PN conducted and supervised the the vaccine trial, supervised and sample collection. KM and PB obtained the funding, and supervised the overall conduct of research. CD was involved in antibody measurements and interpretation of the data. PB conceptualized and provided supervision for the study, supervised and managed the clininical trial, analysed and interpreted the data, prepared the metadata and wrote the paper. All the authors reviewed the manuscript.
