# Supplementary material for: A seven-year study on the effect of the pre-erythrocytic malaria vaccine candidate RTS,S/AS01 E on blood stage immunity in young Kenyan children
Source: Wellcome Open Res. 2019 Mar 5;4:42. [Version 1] doi: 10.12688/wellcomeopenres.15002.1 (PMC6545824; doi:10.12688/wellcomeopenres.15002.1)
Supplement: Peer review history

# Peer review: Reviewer response for version 1

**DOI:** 10.21956/wellcomeopenres.16366.r35552

**Published:** 2019-05-31

**Authors:** Tuan M. Tran

**Affiliations:** 1 Division of Infectious Diseases, Department of Medicine, Indiana University School of Medicine, Indianapolis, IN, USA

General Comments

 In this study, the authors take advantage of a rich collection of longitudinally sampled plasma from an RTS,S malaria vaccine trial to address questions of blood-stage antibody responses after vaccination. In their prior work, they showed that RTS,S vaccinees have reduced antibodies against four merozoite antigens during the 12 months after vaccination (Bejon JID 2013). Here, as a follow-up, they compare antibody levels in RTS,S and control vaccinees at multiple intervals up to 84 months and assess the relationship between Pf-specific antibodies and prospective malaria risk during the interval prior to the following antibody time point.

 Overall, the unadjusted data supports (albeit with modest significance at most time points) the main conclusion that humoral immunity to tested blood-stage antigens is hampered or delayed by RTS,S vaccination, and this reduction in immunity is extended for years after vaccination. As the authors mention in the Discussion, they only test four merozoite antigens that may not elicit naturally protective antibody responses, and so the connection to RTS,S-associated long-term reductions in protective blood-stage immunity still remains a question.

 As shown in their previous work, the differences in antibody responses after vaccination was affected by RTS,S vaccine-induced differences in prior malaria episodes. Thus, it would be important to show if the significant difference between comparisons in Fig 2 and Table 1 still hold after adjusting for prior episodes. Related to this, and to provide more conservative interpretations of their results, if one were to account for multiple comparisons (three comparisons per time point), only EBA175 and MSP1 have significant differences at 24, 36, and 60; and at 8.5, 24, 36, respectively.

 In the Discussion, the statement “In the presence of asymptomatic infection, anti-EBA175 antibodies at all the deciles were higher than the lowest (i.e. nonreactive) decile” is redundant and unclear as written. Was the intention to state “…[malaria protection] for anti-EBA175 antibodies at all the deciles [was] higher…,” given the lower IRR in the higher deciles for EBA175? The subsequent clause, “and some of the higher deciles for AMA1 andMSP3 antibodies were associated with protection from clinical malaria (irrespective of the vaccine arm)” is not supported by the plots in Fig. 3 given most of the 95%CI bars cross unity (the lone exception being the highest decile for AMA1).

 It is notable that the lone antigen that clearly did not show a significant group difference in Fig. 2 was MSP3, which, interestingly, was also the only antigen expressed as FVO, with the others being 3D7. As the authors are aware, the RTS,S vaccine was most effective against the vaccine strain (3D7) (Neafsey et al NEJM 2015). Thus, a possible explanation for the lack of difference for MSP3 is similarity in incidence of malaria episodes caused by non-vaccine strains. This possibility should be added to the discussion. It would be interesting to compare antibody data for 3D7 antigens vs. antigens from heterologous strains, especially a genetically divergent one such as FVO.

 Minor Comments:

 To harmonize with the main text, it would be more useful for the reader if Fig. 1 showed the best fit curve (fitted to standards) for each antigen with sample values superimposed to provide a sense of how many samples were on the linear versus lower and upper plateaus. 

 4 or 5 parameter logistic models are more frequently used for ELISA dilutional standard curves as they most often give the best fit. The authors should briefly mention why they opted for a less conventional model.

 Also, can the authors explain the rationale for using Poisson regression to estimate malaria risk at each interval in this study when Cox regression was used in their previous study? Did some of the underlying assumptions change when the evaluation was extended from 12 m to 84 m?

 Although the timing of the sampling can be somewhat inferred from Fig. 2, Table 1, and the text, it would be helpful for readers if there was a Fig. showing the plasma sampling intervals.

 In the Results section, the authors use "age" in the first reference to Fig. 2. However, Fig. 2 plots use months from the 3rd vaccine dose on the X axes. "Age" should be changed to months after vaccine dose as the latter is more accurate.

 In the statistical analysis section, please indicate that deciles were determined on a per time point basis if this is the case.

 For better readability, semicolons should be used to separate the listed items (months 12, 24, and 36; 24 and 36; and 6, 12, 23, 36, and 84).

 For the AMA1 in Fig. 2, the y axis could be re-scaled to better visualize the significant difference between groups at the circled time points. Currently, lines for both groups appear superimposed.

 Note that that there is a discrepancy in Fig. 2: Text legend states shaded regions are 95% CI whereas Fig. legend denotes these are IQRs. As such, Fig. 2 also needs clarification with regard to which comparisons were means and which were comparisons of medians. If means, then means with 95% CI's should be shown. If medians, then medians with IQR's should be shown. (Typically for comparisons where P<0.05, the 95% CI's of means should not overlap.)

 For Fig. 2, Table I, and text, it appears that month 6 and 8.5 all represent that same time point, which is the March 2008 time point (variable number of days post vaccinations). If this is the case, please use consistent nomenclature to avoid confusion (I suggest March 2008 as this is probably the least ambiguous).

 For Fig. 3, the main text and legend mention deciles were used but only 5 categories are shown for AMA1 and 8 categories for the other 3 antigens. If the lowest deciles (5 for AMA1 and 2 for the others) were combined into a single reference group, please indicate in the legend. Also, it might be clearer if X axis was labeled as categorical deciles (0-10%, 11-20%, etc) as the current X axis can be assumed to be continuous.

 Typographical notes:

 For the last sentence of ELISA methods, extra "s" in “levelss”

 For Fig. 3 legend, "The analysis [was] adjusted for..."

 In first paragraph of Discussion, “MSP1 _24_”

 In Data availability, “Ha[r]vard”

I have read this submission. I believe that I have an appropriate level of expertise to confirm that it is of an acceptable scientific standard, however I have significant reservations, as outlined above.

## References

[1] BejonPCookJBergmann-LeitnerEOlotuALusinguJMwacharoJVekemansJNjugunaPLeachALievensMDuttaSvon SeidleinLSavareseBVillafanaTLemngeMMCohenJMarshKCorranPHAngovERileyEMDrakeleyCJ: Effect of the pre-erythrocytic candidate malaria vaccine RTS,S/AS01E on blood stage immunity in young children. *J Infect Dis*.2011;204(1) : 10.1093/infdis/jir222 9-18 10.1093/infdis/jir222 21628653PMC3105039

[2] NeafseyDEJuraskaMBedfordTBenkeserDValimCGriggsALievensMAbdullaSAdjeiSAgbenyegaTAgnandjiSTAidePAndersonSAnsongDAponteJJAsanteKPBejonPBirkettAJBrulsMConnollyKMD'AlessandroUDobañoCGesaseSGreenwoodBGrimsbyJTintoHHamelMJHoffmanIKamthunziPKariukiSKremsnerPGLeachALellBLennonNJLusinguJMarshKMartinsonFMolelJTMossELNjugunaPOckenhouseCFOgutuBROtienoWOtienoLOtienoKOwusu-AgyeiSParkDJPelléKRobbinsDRussCRyanEMSacarlalJSogoloffBSorghoHTannerMTheanderTValeaIVolkmanSKYuQLapierreDBirrenBWGilbertPBWirthDF: Genetic Diversity and Protective Efficacy of the RTS,S/AS01 Malaria Vaccine. *N Engl J Med*.2015;373(21) : 10.1056/NEJMoa1505819 2025-2037 10.1056/NEJMoa1505819 26488565PMC4762279

# Peer review: Reviewer response for version 1

**DOI:** 10.21956/wellcomeopenres.16366.r35551

**Published:** 2019-05-29

**Authors:** Robin Stephens

**Affiliations:** 1 Department of Microbiology and Immunology, University of Texas Medical Branch at Galveston, Galveston, TX, USA

In order to test the hypothesis that immunity to clinical malaria is delayed by RTS,S, this manuscript details the findings from a study of serum ELISA antibody levels of blood stage antigens found in blood from RTS,S trial participants or Rabies vaccine controls. As this cohort was studied over 7 years, and active surveillance data is available, it is an invaluable indication of the effects of this vaccine on infection, clinical episodes and immune parameters affected. IgG to MSP1-42, (3D7), MSP3 (FVO), PfEBA175RII (3D7), and AMA1 (3D7) were used as an indication of exposure and/or immune status and found to represent (recent) exposure best, though they do accumulate with time since vaccination/age.

 Please describe the extent of exposure of participants using averages or distribution of EIR or episodes. Perhaps the result would depend on this range. All I see is "Exposure Index, as previously described...based on geographical location".

**Fig 1:** I suggest you break the y-axis to show the normal (are they all normal?) part of the curve more clearly. You could label the number of patients represented in the negative fraction on the graph? Is it 500/1735? Is density the correct label for the y-axis, not sure what that means.

**Fig 1 result:** I would say values below the "linear part of the sigmoid curve" of the hyperimmune standard curve, were considered unresponsive. Are these the data points marked as a concentration of 2 in Figure 1?

**Fig 2 text:** The increase with age is apparent for AMA1 for participants with highest levels, the difference may be in the sensitivity of the ELISA.

**Fig 2: **The legend could be more descriptive of the data and less of the method. Define IQR, CTl vs Vac in legend. Also, more could be done to make the figure print well in black and white-change shape of symbols and lines in one group. Could significance be indicated on the graph itself?

**Table 1:** Text - the sentence describing the data is hard to understand. "Antibody levels for A, E, M diverged after vaccination. Levels were higher for Rabies vaccinees than RTS,S vaccinees for AMA1 at 24 months, for EBA at 24-72 months, and for MSP1 8.5, 24, 36 and 84 months. (Unless p<.05 is not the cutoff?) But as is, only two sets of dates are listed with three antigens listed "respectively". The table itself could be improved by adding stars for significance, and by separating antigens with a thicker line for easier readability.

**Figure 3:** Great analysis, please state the meaning of IRR-Incident Rate Ratio in the legend, or on the graph.

 Can you tell from your data, if the larger error bars for MSP1 slide positive group, are due to the faster decline of these antibodies compared to other specificities? It will be great when robust  neutralizing assays have been developed that could distinguish effective blood stage antibody from ELISA positive/exposure induced.

 In the last sentence, maybe you mean among the children with asymptomatic parasitemia? or without symptomatic parasitemia?

**Discussion:** Please check the run-on sentence "In the presence of asymptomatic infection, anti-EBA175 antibodies at all the deciles were higher than the lowest (i.e. non-reactive) decile (I'm already lost), and some of the higher deciles for AMA1...".

 I would add a sentence about the positive side-reduced exposure leading to reduced clinical episodes. How does that balance out with the potential for increased episodes from delayed immunity, are they predicted to be worse episodes, as in later severe disease?

I have read this submission. I believe that I have an appropriate level of expertise to confirm that it is of an acceptable scientific standard.

# Peer review: Reviewer response for version 1

**DOI:** 10.21956/wellcomeopenres.16366.r35016

**Published:** 2019-03-18

**Authors:** Wilson L. Mandala

**Affiliations:** 1 Malawi Liverpool Wellcome Trust Clinical Research Programme, Biomedical Sciences Department, College of Medicine, Blantyre, Malawi; 2 Malawi University of Science and Technology, Thyolo, Malawi

This is an extension of a previously published work (Bejon *et al.*, 2011 ^1^) on the effect of the RTS,S vaccine of the development of the natural immunity against blood stage malaria. The authors have previously reported some reduction in antibody titers against specific *P. falciparum* antigens. What did not come up clearly in this previous report was the longevity of this RTS,S-related effect. This recent report therefore was aimed at addressing that point.

 Some major points:

1. There are some discrepancies in the presentation of the time points mentioned in the Methods section [0, 4, 6.5, 8, 14, 24, 36, 48, 60, 72 and 84 months] whereas what is being presented in the Results section (Table 1) seems to have slightly different time points [0, 4, 8.5, 12, 24, 36, 48, 60, 72, 84]. This needs to be checked and the correct time points used in both sections and throughout the manuscript.
2. The authors state that the geometric means of antibodies against the four *P. falciparum* antigens were lower in the RTS,S group compared to the group that had the Rabies vaccine administered. Going through Table 1 the trend seems to be different from this. The trend shown in Figure 2 does agree with the authors’ report but not with the trend observed in Table 1. The authors therefore need to clarify how the results presented in Table 1 differ from those presented in Figure 2.
3. The statement “antibodies to four specific merozoite antigens were not associated with clinical protection, rather anti-merozoite antibodies were positively associated with risk of clinical malaria for the group as a whole.” is a strong and potentially damaging report which does not augur well with the overall aims of out-rolling this vaccine candidate. It might be considered to water down the whole point of introducing this vaccine and may need to be revisited and presented in a more promising manner. More importantly, the authors seem to contradict this strong statement with the follow up statement: “it is possible that higher antibodies titers might have been protective (i.e. those above a protective threshold)." To marry these two rather disjointed/conflicting statements the authors may wish to start off by introducing the results and proposed concept emanating from this study reported by Kinyanjui *et al.* (2004 ^2^) and say more about this proposed threshold of antibody titers above which they confer protection against malaria infection but below which they do not. They can then proceed by stating if the antibody titers detected in this study are either above or below that threshold level and then proceed to speculate if there is merit on the statement that the administration of the RTS,S vaccine directly (or indirectly) impairs the development of the P.f. specific antibodies. It’s worth noting that it is the same team who reported a 53% efficacy for the RTS,S vaccine with 38 episodes of clinical malaria in the RTS,S arm compared to 89 episodes observed in the control group vaccinated with a Rabies vaccine that was associated with higher anticircumsporozoite antibody titers (Bejon *et al.,* 2008 ^3^). This detail needs to come in their discussion.

Minor comments:

1. The authors need to justify the choice of Rabies vaccine in the control group either by referring to their previous works or other related works.
2. There is need for a paragraph on the limitations of the study in the Discussion section.
3. Their previous paper on the same Kenyan cohort combined data from Kenyan and Tanzanian participants. It’s worth mentioning why this time they have decided to report only on the Kenyan cohort.
4. The observation that antibody levels do not correlate positively with immunity against malaria has been discussed in detail in a number of good papers (Fowkes *et al.*, 2010 ^4^, Osier *et al.*, 2008 ^5^, Osier *et al.*, 2014 ^6^, Stanisic *et al.*, 2015 ^7^ and Crompton *et al.*, 2010 ^8^). The authors might wish to refer to these in their introduction and discussion sections.
5. The authors raise the point about malaria transmission control measures and vaccine approaches being effective in controlling the pre-erythrocytic stages of the malaria infection but, not only failing to confer robust antibody mediated immunity, but might even be compromising/impairing the development of the P.f. antibody-mediated immunity against the blood stage in the affected children. Maybe they need to point out how a combination of these measures might have a compounded effect on the development of merozoite-stage immunity.
6. In the discussion section the authors propose that the administration of the RTS,S vaccine should be combined with the provision of other malaria control measures like ITNs. They also suggest that the four-dose vaccine regimen should include long-term follow up in the implementation trials. Adding a paragraph on informed speculations on what could be achieved by combinations of vaccine candidates (especially one specific for the sporozoite stage and one specific for the merozoite stage) might add weight to their argument. This is in light of their observation that RTS,S might actually be predisposing the recipient children to acquiring blood stage malaria infection presumably due to the impaired development of this stage-specific immunity.
7. They only introduce about the four-dose in passing in the discussion section. Could they please expand on that and say more on what is being proposed to be implemented?
8. The fact that seasonality was not entirely eliminated as a potential confounded in this second report needs to be expanded in the discussion section with appropriate reference to how seasonality was observed to affect the outcome of the vaccine in the previous study/report.
9. In the introduction please consider change the sentence to “...based on the circumsporozoite protein (CSP) that targets the pre-erythrocytic STAGE (not cycle) of the P. falciparum life cycle in humans.”
10. The main objective was to determine the duration of the effect of the RTS,S vaccine. Has this been achieved and reported appropriately? Could they do this better by referring to what they had reported in their JID paper (Bejon *et al.*, 2011 ^1^) and build on that in this report?
11. Since they are using pool serum samples from healthy controls, could the authors clearly state how blood samples were collected from the study participants, what volume, in what tubes, and what anticoagulant was used?  
12. The plasma samples were collected at different stages of the seven year duration implying some were stored for longer than others. What measures were put in place to account for a possible deterioration of the antibodies which could account for the reported decline of antibody titers with time?
13. The legend for Figure 1: “Distribution of anti-merozoite antibody levels. Antibody levels were measured by ELISA” requires more details.
14. The legend for Table 1 needs to be revised and delete “of antibody levels” as this sounds like a repetition.
15. In Figure 3, the Y-axis label (IRR) needs to be written in full either in the legend or on the axis.

I have read this submission. I believe that I have an appropriate level of expertise to confirm that it is of an acceptable scientific standard, however I have significant reservations, as outlined above.

## References

[1] BejonPCookJBergmann-LeitnerEOlotuALusinguJMwacharoJVekemansJNjugunaPLeachALievensMDuttaSvon SeidleinLSavareseBVillafanaTLemngeMMCohenJMarshKCorranPHAngovERileyEMDrakeleyCJ: Effect of the pre-erythrocytic candidate malaria vaccine RTS,S/AS01E on blood stage immunity in young children. *J Infect Dis*.2011;204(1) : 10.1093/infdis/jir222 9-18 10.1093/infdis/jir222 21628653PMC3105039

[2] KinyanjuiSMHowardTWilliamsTNBullPCNewboldCIMarshK: The use of cryopreserved mature trophozoites in assessing antibody recognition of variant surface antigens of Plasmodium falciparum-infected erythrocytes. *J Immunol Methods*.2004;288(1-2) : 10.1016/j.jim.2004.01.022 9-18 10.1016/j.jim.2004.01.022 15183081

[3] BejonPLusinguJOlotuALeachALievensMVekemansJMshamuSLangTGouldJDuboisMCDemoitiéMAStallaertJFVansadiaPCarterTNjugunaPAwuondoKOMalabejaAAbdulOGesaseSMturiNDrakeleyCJSavareseBVillafanaTBallouWRCohenJRileyEMLemngeMMMarshKvon SeidleinL: Efficacy of RTS,S/AS01E vaccine against malaria in children 5 to 17 months of age. *N Engl J Med*.2008;359(24) : 10.1056/NEJMoa0807381 2521-32 10.1056/NEJMoa0807381 19064627PMC2655100

[4] FowkesFJRichardsJSSimpsonJABeesonJG: The relationship between anti-merozoite antibodies and incidence of Plasmodium falciparum malaria: A systematic review and meta-analysis. *PLoS Med*.2010;7(1) : 10.1371/journal.pmed.1000218 e1000218 10.1371/journal.pmed.1000218 20098724PMC2808214

[5] OsierFHFeganGPolleySDMurungiLVerraFTettehKKLoweBMwangiTBullPCThomasAWCavanaghDRMcBrideJSLanarDEMackinnonMJConwayDJMarshK: Breadth and magnitude of antibody responses to multiple Plasmodium falciparum merozoite antigens are associated with protection from clinical malaria. *Infect Immun*.2008;76(5) : 10.1128/IAI.01585-07 2240-8 10.1128/IAI.01585-07 18316390PMC2346713

[6] OsierFHMackinnonMJCrosnierCFeganGKamuyuGWanaguruMOgadaEMcDadeBRaynerJCWrightGJMarshK: New antigens for a multicomponent blood-stage malaria vaccine. *Sci Transl Med*.2014;6(247) : 10.1126/scitranslmed.3008705 247ra102 10.1126/scitranslmed.3008705 25080477PMC4856877

[7] StanisicDIFowkesFJKoinariMJavatiSLinEKiniboroBRichardsJSRobinsonLJSchofieldLKazuraJWKingCLZimmermanPFelgerISibaPMMuellerIBeesonJG: Acquisition of antibodies against Plasmodium falciparum merozoites and malaria immunity in young children and the influence of age, force of infection, and magnitude of response. *Infect Immun*.2015;83(2) : 10.1128/IAI.02398-14 646-60 10.1128/IAI.02398-14 25422270PMC4294228

[8] CromptonPDKayalaMATraoreBKayentaoKOngoibaAWeissGEMolinaDMBurkCRWaisbergMJasinskasATanXDoumboSDoumtabeDKoneYNarumDLLiangXDoumboOKMillerLHDoolanDLBaldiPFelgnerPLPierceSK: A prospective analysis of the Ab response to Plasmodium falciparum before and after a malaria season by protein microarray. *Proc Natl Acad Sci U S A*.2010;107(15) : 10.1073/pnas.1001323107 6958-63 10.1073/pnas.1001323107 20351286PMC2872454
